# Neural indices of listening effort in noisy environments

**DOI:** 10.1038/s41598-019-47643-1

**Published:** 2019-08-02

**Authors:** Andrew Dimitrijevic, Michael L. Smith, Darren S. Kadis, David R. Moore

**Affiliations:** 1 Department of Otolaryngology, Head and Neck Surgery, Sunnybrook Health Sciences Centre, Toronto, ON Canada; 2 Department of Otolaryngology, Head and Neck Surgery, University of Toronto, Toronto, ON Canada; 3 Communication Sciences Research Center, Cincinnati Children’s Hospital Medical Center, Cincinnati, USA; 4 Division of Neurology, Cincinnati Children’s Hospital Medical Center, Cincinnati, OH USA; 5 Pediatric Neuroimaging Research Consortium (PNRC), Cincinnati Children’s Hospital Medical Center, Cincinnati, OH USA; 6 College of Medicine, Department of Pediatrics, University of Cincinnati, Cincinnati, OH USA; 7 Department of Otolaryngology, College of Medicine, University of Cincinnati, Cincinnati, OH USA; 8 Manchester Centre for Hearing and Deafness, University of Manchester, Manchester, UK; 9 Department of Speech and Hearing Sciences, University of Washington, Seattle, WA USA

**Keywords:** Cortex, Language, Perception

## Abstract

Listening in a noisy environment is challenging for individuals with normal hearing and can be a significant burden for those with hearing impairment. The extent to which this burden is alleviated by a hearing device is a major, unresolved issue for rehabilitation. Here, we found adult users of cochlear implants (CIs) self-reported listening effort during a speech-in-noise task that was positively related to alpha oscillatory activity in the left inferior frontal cortex, canonical Broca’s area, and inversely related to speech envelope coherence in the 2–5 Hz range originating in the superior-temporal plane encompassing auditory cortex. Left frontal cortex coherence in the 2–5 Hz range also predicted speech-in-noise identification. These data demonstrate that neural oscillations predict both speech perception ability in noise and listening effort.

## Introduction

We live in complex noisy environments. Typical human interactions occur in places where we need to focus our attention on one talker while ignoring others. Anecdotally, most of us have experienced some degree of listening effort following a conversation in a noisy pub and have noticed that we need to expend a lot of “cognitive effort”^1,2^ to successfully follow the conversation. The neural mechanisms of this “listening effort” are not well understood. To date, the study of listening effort has mostly focussed on indirect measures of brain activity (e.g., arousal mediated pupil diameter). In this study we describe a direct, brain-based biomarker of listening effort that is mediated through left frontal language areas of the brain and sensory regions of the auditory cortex. We chose to study people who are hearing impaired and known to have greater degrees of listening effort compared to normal hearing listeners^3,4^. Increased effort has been related to increased frustration, fatigue, and decreased concentration in this population, impacting work or school performance, resulting in overall poor quality of life, social withdrawal and depression^5,6^. The current philosophy in hearing loss management is to restore to sound frequency thresholds to normal ranges with no regard to manage how hard it is to listen.

Research on the effects of listening effort on hearing is still in its infancy. One challenge is to quantify listening effort. The current battery of objective tests of listening effort includes pupil diameter (pupillometry), dual-task measures, where listening effort is inferred from reductions in secondary task performance while the primary task is a listening task such as speech in noise perception; see review in Gagné^7^, or reaction times while listening in a challenging environment (see review in McGarrigle^1^). Although there is a rich history of using these methods to quantify listening effort, they are indirect measures of brain activity. Having a brain measure of listening effort in hearing impaired populations has far reaching implications. If the site(s) of dysfunction leading to increased listening effort can be inferred from brain physiology, clinicians could use this to guide optimal rehabilitation, for instance focussing on cognitive intervention in conjunction with hearing technology. Cognitive factors including executive function, working memory, attention and motivation are thought to be strongly engaged during effortful listening^2^. A recent meta-analysis of brain imaging studies (fMRI, functional magnetic resonance imaging and PET, positron emission tomography) suggested that effortful listening involves the recruitment of brain regions beyond primary auditory cortex^8^. Speech-in-noise typically recruited left inferior frontal gyrus (IFG), left inferior parietal lobule, and right insula. Effort resulting from spectral degradation typically recruited insula bilaterally and the left superior temporal gyrus (STG). Effort involving linguistic complexity typically activated the left IFG, right middle frontal gyrus, left middle temporal gyrus and bilateral STG. The common feature of these studies is that effortful listening was associated with recruitment of primary auditory cortex, the canonical language network (left perisylvian regions, including Broca’s and Wernicke’s areas), and extracanonical regions involved in language processes^9^.

A limited number of electroencephalogram (EEG) studies have addressed neurophysiological correlates of listening effort. Wisniewski and colleagues^10^ employed a speech in noise measure (English Sentence Matrix test) while participants reported subjective ratings of effort. Effort was correlated with an increase in frontal theta (4–7 Hz) activity. Wöstmann and colleagues^11^ observed that alpha power (7–13 Hz) varied proportionately with self-reported listening effort (not related to the stimuli that were used in the study) in a listening task that altered speech spectral detail. We^12^ and others^13^ have shown that listening in challenging environments (e.g., speech in noise or vocoded speech) is associated with more alpha modulation, especially in parietal cortex, relative to less challenging tasks. The mechanism underlying an increase in alpha power is incompletely understood and an increase in parietal alpha may be related to inhibition of competing visual inputs^14^ or inhibition of dorso-ventral attention pathways enhancing speech object processing^12^. Decreases in temporal-lobe alpha, related to auditory cortex activation, have been observed during speech perception in noise and were a better predictor of speech identification than parietally-generated alpha^12^.

Measures of neural entrainment to speech and other auditory signals may also provide insight to the mechanisms of listening effort. Neural entrainment, also referred to as ‘neural tracking’ or ‘cerebro-acoustic coherence’, is a measure that relates acoustic feature fluctuations to brain activity fluctuations. The simplest rhythmic feature is sinusoidal amplitude modulation giving rise to auditory steady-state responses^15^, defined as a peak in the EEG amplitude spectrum at the amplitude modulation rate. More recently, research focus has shifted towards natural speech, where the amplitude envelope^16–19^ or phonetic information^20^ is related to brain fluctuations. Neural entrainment to speech envelopes has been associated with successful selective attention in dichotic listening tasks^21–23^ but has never been used to quantify the degree of listening effort. Here, we also demonstrate that the degree of neural entrainment to speech envelopes is related to speech perception in noise and to listening effort arising from different areas of the brain.

We examined the relationship between alpha oscillations, neural entrainment and listening effort in cochlear implant (CI) users. A CI is a prosthetic device that delivers auditory stimulation via electrodes surgically implanted in the cochlea^24^. The incoming sound is received via a microphone behind the ear and processed in a series of filter banks. The filtered sound envelope is used to modulate an electrical pulse train that stimulates the auditory nerve. The representation of the sound is degraded in both spectral and temporal domains. The spectral degradation is partly due to the reduced number of channels or stimulating electrodes and to the large current spread to other electrodes resulting in broader psychoacoustic filters compared to normal acoustic hearing^25^. This degradation results in reduced speech in noise perception^26^ and reduced perceptual segregation between target and masker stimuli^27^. Temporal processing degradation in CI users has often been quantified using amplitude modulation detection thresholds related to speech perception ability^28,29^. Reduced spectro-temporal encoding ability in CI users may lead to recruitment of extra cognitive resources to understand speech in noise. CI users typically require more effort while listening to speech in noise compared to normal hearing counterparts^3,4^, even when the intelligibility of the speech is adjusted for equal performance^4^.

In this study we used a speech-in-noise procedure (the ‘digits-in-noise’; DIN) that is becoming widely used^30^ and is closely related to pure tone thresholds in people with hearing impairment^31^. Previously, we found that EEG alpha oscillations in normal hearing listeners are modulated by attention and accuracy in performing the DIN^12^. Here we demonstrate that listening effort during DIN testing in CI users is related to: (1) increased alpha oscillations in left frontal regions, similar to fMRI activation in normal hearing^8,32^ and, (2) decreased neural entrainment to the speech envelope of the DIN stimulus. The general pattern of oscillatory activity is the same in both normal hearing listeners^12^ and CI users in this study, therefore we believe that these findings are not limited to just CI users but rather generalizable to the normal hearing population.

## Results

### Attention modulates brain oscillations in CI users

All the DIN stimuli for the electrophysiological recordings were presented at a digit signal-to-noise ratio (SNR) corresponding to the individual’s speech reception threshold (SRT). The SRT is the digit signal-to-noise ratio yielding 50% correct performance^12^. The SRT was determined in each participant in a prior behavioural session, before the EEG DIN data were collected. CI users had behavioural SRTs ranging from −6 to −13 dB (mean −7.7 dB; SD 2.7). This is significantly (t(21) = −10.8; p < 1e-6) greater than the SRTs (mean = −16.4 dB, SD = 0.8) observed in our previous DIN study in normal hearing listeners^12^ confirming that CI users have lower performance perceiving speech in noise.

**Figure 1:**
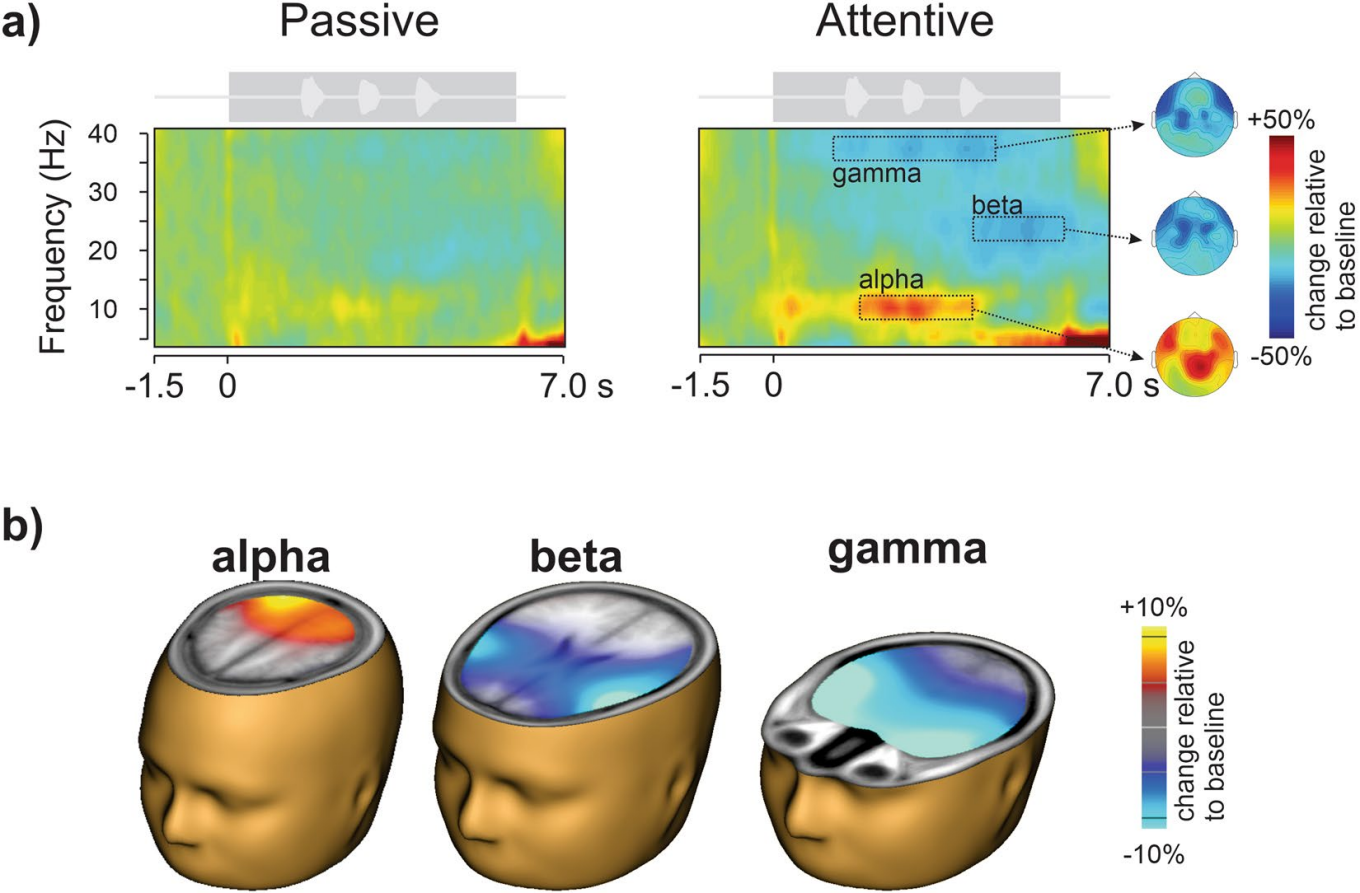
Attention enhances brain oscillations. (**a**) Grand mean time-frequency representations for the passive (left) and attentive (right) listening conditions. The digits in noise acoustic waveform is shown above each plot (light grey) indicating the timing of the stimuli relative to the time-frequency plot. The darker grey rectangle schematically shows the noise onset and offset. Oscillatory power increased (red; event-related synchronization, ERS) or decreased (blue; event-related desynchronization, ERD) as a percent change relative to baseline (−1 to 0 s). The attentive condition was characterized by greater oscillatory power change in the alpha (8–12 Hz) band, and reduced power in the beta (20–28 Hz) and gamma (35–40 Hz) bands compared to the passive listening condition. Time frequency data were averaged across all 63 electrodes. Oscillatory topography is shown on the right. (**b**) Beamformer source analysis over the dotted time-frequency window indicated that the alpha ERS is generated in the right posterior parietal cortex, beta ERD in left inferior frontal gyrus, and gamma ERD in bilateral anterior poles. Head and brain images were in generated in BESA Research 7.1 (http://www.besa.de/).

CI users listened to DIN in both passive and attentive listening conditions, where attentive listening required verbal identification of the presented digits. During passive listening, CI users were instructed to ignore the digits while they watched a closed-captioned movie of their choice. Figure 1 shows the grand mean time-frequency representations across all CI users during passive and attentive listening conditions. The most prominent differences in response to the two listening conditions was the increase (or event-related synchronization; ERS^33^) in alpha power (8–12 Hz) during digit presentation (Fig. 1a), bursts of event-related desynchronization (ERD) for gamma (35–40 Hz), synchronized with each of the presented digits, and a beta (20–28 Hz) ERD at final digit offset. The scalp topography indicated a centroid over parietal for the alpha ERS and frontal and central peaks for the beta and gamma. Beamformer^34^ source analysis was performed for each participant based on the maximum ERD/ERS time-frequency windows in the grand mean data. The dominant generators were the right parietal cortex for alpha, left IFG for beta, and bilateral anterior temporal lobes for gamma (Fig. 1b).

### Left frontal alpha power predicts listening effort

**Figure 2:**
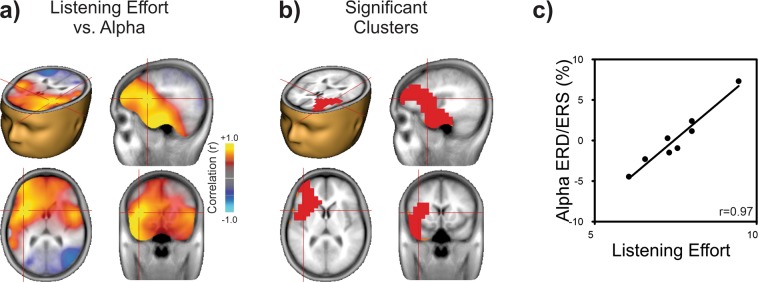
Listening effort is related to alpha power in left frontal regions. (**a**) Correlation between alpha power (for each beamformer voxel) and listening effort (NASA Task Load Index rating from 1–10) rating across all CI users. Each voxel represents a Pearson correlation coefficient. Yellow represents voxels with high positive correlations and blue represents high negative correlations. The maximum positive correlation is indicated by the cross hairs at Talairach location −39, 11, 10 corresponding to Brodman area 44, the left inferior frontal gyrus (IFG). (**b**) Only the left frontal and temporal clusters survived multiple comparisons corrections (p = 0.048, corrected). (**c**) A scatter plot between listening effort and alpha power at the left IFG voxel with correlation value of r = 0.97. Increased effort is associated with increased alpha ERS. Head and brain images were in generated in BESA Research 7.1 (http://www.besa.de/).

Listening effort was assessed using the NASA Task Load Index^35^ after each recording block. This self-reported rating was then used to perform the correlations with the electrophysiological data offline. Figure 2 shows that alpha power during the DIN task is significantly correlated with self-reported effort for the digit identification task. Correlations close to r = +1 were observed bilaterally in frontal regions encompassing left inferior frontal gyrus (IFG) and insula (Fig. 2a; peak at Talairach −39, 11, 10 coordinates, IFG according to http://sprout022.sprout.yale.edu/mni2tal/mni2tal.html). Significant correlation clusters were observed in these regions after multiple comparisons corrections^36^ (Fig. 2b; criterion: p = 0.048) CI users with higher alpha ERS showed greater listening effort whereas those with greater alpha desynchronization (ERD) showed less listening effort (Fig. 2c). No significant correlations were observed between alpha power source and DIN identification performance. Note that although the dominant alpha ERS source was found to be in parietal regions (Fig. 1), the peak correlations between alpha power and listening effort were observed in left frontal regions. This apparent brain source discrepancy is a result of using different computational approaches for the two measures. Parietal alpha ERS was computed from the peak activity for the beamformer source localization across subjects whereas the left frontal alpha listening effort was the result of computing correlations between individual subject’s alpha source and listening effort ratings, yielding a Pearson correlation value for each brain voxel. The left frontal regions showed the most consistent alpha relationship with listening effort even though the source was dominated by parietal regions.

No significant correlations between beta or gamma sources and listening effort were observed. However, interestingly, a negative correlation (r = −0.93) between gamma power and digit identification was observed in the occipital cortex (data not shown).

### Brain coherence with the speech envelope is related to listening effort

**Figure 3:**
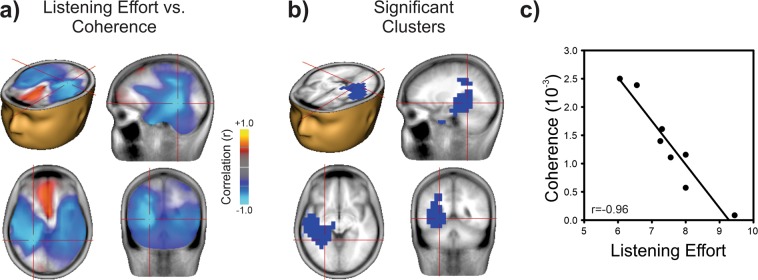
Listening effort is related to speech-brain coherence. (**a**) Correlation between listening effort and speech-brain coherence (in the 2–5 Hz range) across all CI users. Each voxel represents a Pearson correlation coefficient, as in Fig. 2. High negative correlations were observed bilaterally in the temporal lobe, including auditory cortex. The minimum correlation is indicated by the cross hairs at Talairach location -25, -45, -4 corresponding to Brodman area 37, the left fusiform gyrus. (**b**) Only the left temporal clusters survived multiple comparisons corrections. (p = 0.039 for cluster). (**c**) Relation between listening effort and speech-brain coherence power measured at the peak correlation (left fusiform gyrus voxel with correlation value of r = −0.96). Increased effort is associated with decreased speech-brain coherence. Head and brain images were in generated in BESA Research 7.1 (http://www.besa.de/).

Neural entrainment to the digit speech envelope was assessed using Dynamic Imaging of Coherent Sources (DICS^34^) as implemented in BESA^37^. DICS calculated the coherence between the envelope of the DIN stimuli (low pass filtered at 10 Hz) and neural sources in the 2–5 Hz range over a time range that encompassed the digit onset and offset (1–5 seconds). The envelope of typical human syllables also occurs over the 2–5 Hz range^38^. Figure 3 shows that lower speech-brain coherence was associated with higher listening effort. Correlations between the voxel-wise DIN envelope coherence and listening effort were computed across subjects. Negative correlations approaching r = −1 were observed bilaterally in the temporal lobe encompassing the auditory cortex (Fig. 3a). Only left hemisphere clusters survived corrections for multiple correlation comparisons (Fig. 3b). As with the alpha source described above, correlations between DIN identification and coherence were not significant.

### Brain coherence in left frontal regions predicts DIN performance

**Figure 4:**
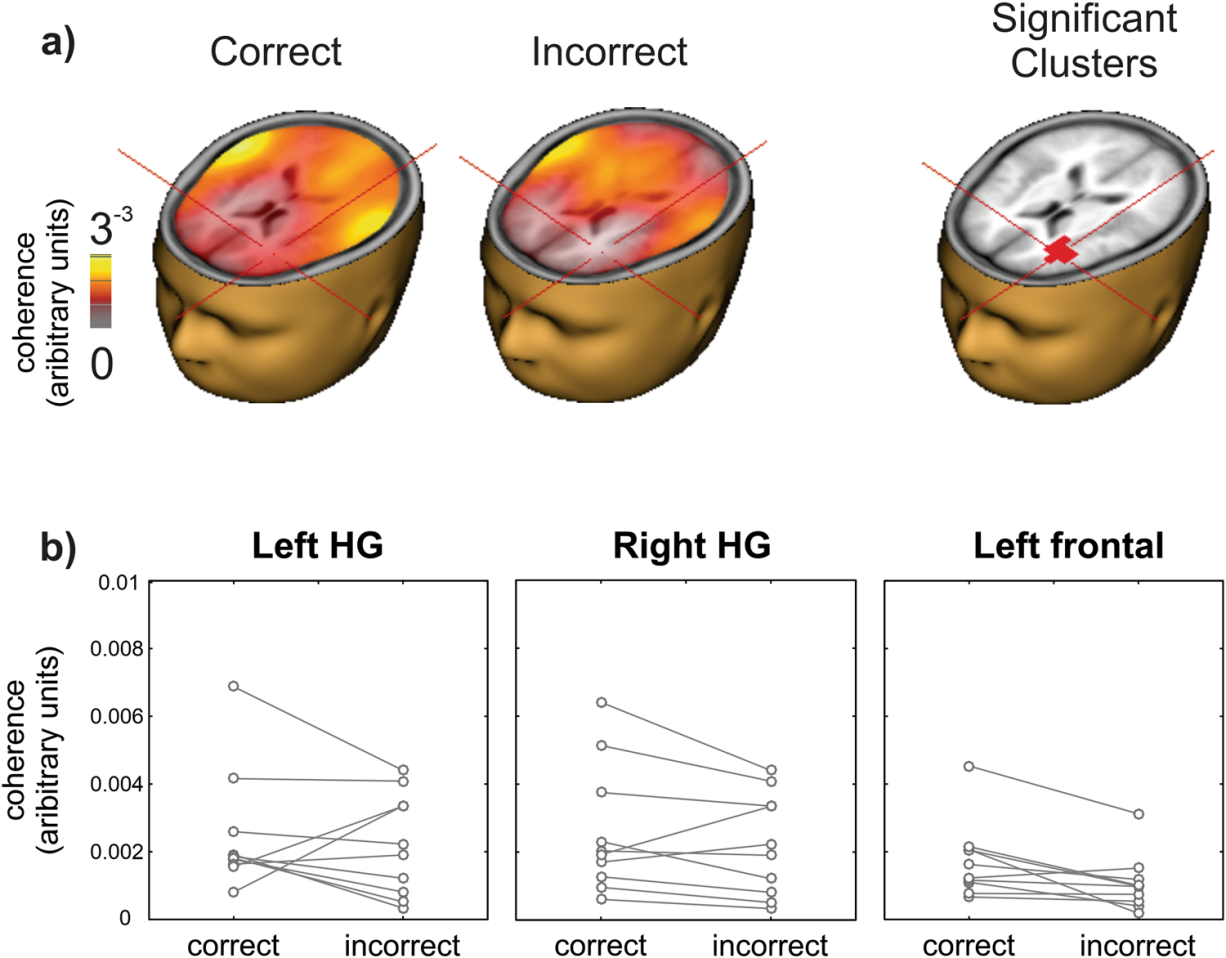
Correct digit identification is related to DIN speech-brain coherence in left frontal regions. (**a**) DICS (DIN speech envelope – to whole brain at 2–5 Hz) were performed separately on correct and incorrect trials. Significant differences between correct and incorrect trials were observed in left prefrontal cortex (cross hairs at −32, 40, 16; p = 0.005). (**b**) Individual coherence values at voxel locations at left and right Heschl’s Gyrus (HG) and at left prefrontal cortex (cross-hairs in (**a**)) for correct and incorrect trials. Only the left frontal pole showed a significant difference between correct and incorrect DIN identification. Head and brain images were in generated in BESA Research 7.1 (http://www.besa.de/).

Speech-brain coherence was compared between trials with correct and incorrect digit identification (Fig. 4). The CI user had to identify all three digits correctly for a trial to be classified as “correct”. Although high coherence in left and right auditory cortex regions on both correct and incorrect trials was observed, a paired-test yielded differences between correct and incorrect identification only in the left prefrontal cortex (criterion: p = 0.005, corrected for multiple comparisons; Talairach coordinates −32, 40, 16; BA10) where higher speech-brain coherence was observed on correct trials. Additionally, Supplemental Fig. S1 demonstrates significant coherence with the speech envelope in left auditory temporal regions.

## Discussion

The purpose of this study was to discover functional and structural neural substrates of self-reported measures of listening effort. Three novel findings were observed: (1) left frontal IFG alpha power was positively associated with listening effort; (2) speech envelope auditory cortex coherence in the 2–5 Hz range was negatively associated with listening effort; (3) accuracy of speech identification was positively associated with envelope speech-brain coherence in the left frontal cortex.

The term ‘listening effort’ was coined by translational auditory scientists to describe phenomena of ‘tiredness’ and the ‘compensation’ required to listen when hearing impaired or using hearing prosthetics. This study identifies neural correlates associated with these phenomena. More recently, focus has shifted to a recognizably cognitive approach. The observed correlation between left IFG alpha activity and self-report listening effort is consistent with a recent meta-analysis report summarizing the brain regions associated with effortful listening^8^. When listening in challenging environments, the classical auditory regions of the superior temporal gyrus (STG) cannot effectively process the speech sound, although they are activated^39^ and cortical regions supporting executive function, attention, memory and sensorimotor processing are recruited^32^. With effortful listening, the most commonly observed activated regions of the brain include the frontoparietal network including left IFG and left inferior parietal lobule. The left IFG includes canonical language production regions such as Broca’s area, known to be activated during covert or internalized speech production^40^ and working memory involving speech^41^. Increased alpha power in a cortical region likely reflects decreased neural activation^42^ by suppressing non-crucial or potentially distracting information allowing for more efficient neural processing. Increases in alpha have, for example, been reported for ipsilateral spatial attention to auditory^43–49^, visual^50^ and somatosensory^51^ stimuli and for modality switching of attention^52^. In this study we observed a continuum of alpha event-related activation, with more ERD for lower effort and more ERS for higher effort ratings. The interpretation of the direction of this alpha-listening effort (ERD, lower effort; ERS higher effort) effect is difficult. Alpha ERD in the left IFG would suggest neural activation with less listening effort. This is opposite to what we would expect given that this increases in this region are observed with greater listening effort^8^. Opposite alpha effects were observed when speech stimuli were used as a distractor stimuli in an auditory working memory study, leading the authors to suggest that the listener’s goal, rather than acoustic degradation, drives increases in alpha^53^. In the present study, the increased alpha may be related to attentional modulation of the distracting noise. Another interpretation is that increased listening effort is manifested as suppression of language production, given that the task involved a verbal response. This may be related to the observation that poor speech SNRs recruit speech motor systems as a compensatory mechanism for speech in noise perception^54^. Nonetheless, the involvement of canonical language areas appears to be involved in the current listening effort paradigm.

A limited number of previous studies have examined neural correlates of listening effort. Our data complement and extend the suggestion by Wöstmann and colleagues^11^ that alpha oscillations are related to listening effort. In their study, normal hearing listeners, young and elderly, had to make predictive number sequence judgments to filtered speech digits. Listening effort in their study was quantified in a general post experiment questionnaire unrelated to the experimental task in time or content. General self-reported listening effort was found to be significantly related to a derived measure of alpha that was modelled on how alpha changes with different acoustic manipulations and stimulus predictability. In the current study a direct relationship between alpha oscillations and listening effort was observed and localized with brain source analysis. We related alpha power during the time participants were listening to the digits in noise to effort ratings of the digits after each block, thereby demonstrating a more direct relationship. The other most relevant study is that from Wisniewski and colleagues^10^ who found that frontal theta was correlated to sentences presented in noise. This effect was attributed to the presumed role that theta plays in working memory. Although there was alpha ERS during sentence presentation in the data of Wisniewski and colleagues^10^ (see Fig. 2 in their study), the relationship with listening effort was not reported. One possible explanation for the differences in theta activity between this study and that of Wisniewski and colleagues is that listening to sentences involves greater demands on working memory than does listening to three digits, therefore yielding more robust theta oscillations. In a series of combined EEG and pupillometry studies, McMahon and colleagues^13,55^ failed to find relationships between alpha oscillations and listening effort. One possible explanation for the discrepancy between the current study and McMahon’s is related to how the alpha oscillations were quantified. Here, correlations across voxels in brain space were measured, whereas in the McMahon studies, observations were limited to parietal sensors. Although we and others have shown that alpha has a dominant generator in the parietal cortex, the relationship with listening effort appears to be left frontally-generated.

In contrast to the alpha oscillations, beta and gamma oscillations showed no relationships with listening effort. The observed beta ERD in left IFG is consistent previous left IFG beta ERD in expressive language paradigms^56,57^ suggesting that at the offset of the digits, speech production is initiated. Although distinct bursts of gamma ERD occurred with digit presentation and localized to anterior temporal lobes, the functional significance of these are difficult to interpret.

In this study we observed two novel speech-brain coherence effects: the strength of speech-brain coherence in auditory cortex was inversely related to listening effort, and speech-brain coherence in left frontal regions was positively related to correct digit identification. Speech-brain coherence is presumably reduced by the addition of energetic noise masking, which poses a particular challenge for CI users^58^. Previous work suggests that how well the auditory neural system entrains to speech is a good predictor of speech perception in noise in normal hearing^59^ and in hearing impaired listeners using a hearing aid^22^. We hypothesized that auditory cortex coherence to the speech envelope would be inversely related to number of correctly identified digits. No such relationship was observed. Rather, a significant inverse relationship with listening effort was found, suggesting that degree of sensory disruption in the auditory cortex from the noise masker is associated with a proportional increase in listening effort. This observation is consistent with previous work showing that reducing the SNR of speech is associated with increased listening effort, as measured with pupillometry^60^. Both frontal IFG and auditory cortex were associated with listening effort and therefore would suggest a functional relationship exists between auditory cortex and left IFG. Although it may be tempting to infer that reduced auditory cortex sensory encoding is driving the left IFG, connectivity measures between alpha rhythms and speech-brain coherence are difficult given that they quantify different types of brain activity. However, recent work examining connectivity measures between left frontal regions and left auditory cortex in the delta/theta (1–7 Hz) range, typical of speech acoustics, have indicated that left frontal regions are a top-down driver for the auditory cortex during natural speech^61^. Functional connectivity from left IFG to auditory cortex has been shown to be stronger in listeners with higher QuickSIN performance^62^. Additionally, frontal-auditory connectivity, seen in both structural and electrophysiological measures, appears to relate to the ability to learn new words, which may be especially important in adults learning to hear again and/or adapt to a cochlear implant^63^. These findings therefore suggest that correct digit identification is associated with left frontal speech-brain coherence driving auditory cortex. Left frontal speech-brain coherence is also consistent with previous fMRI/ECoG (electrocorticography) noise vocoding and sine-wave speech learning studies demonstrating increased left IFG activity after successful speech vocoder training^64–67^.

Although we have described some novel neural metrics for listening effort in cochlear implant users, some study caveats exist. Given that our experimental task involved a verbal feedback of the digits, language production areas of the brain will necessarily be recruited. A non-verbal task would help clarify the role of IFG in listening effort. Additionally, the alpha and coherence correlations with listening effort across subjects were based on a relatively small sample size. Studies with larger samples sizes and with different types of hearing loss are warranted.

Having direct neural correlates of listening effort in CI users has important clinical implications. Pupillometry, dual-task, and reaction time, used in the majority of studies^6^, are indirect measures associated with neural processing. One advantage of auditory electrophysiology, a direct measure of neural activity, is that the location of a disorder can be approximated. For example, with auditory neuropathy, absent auditory brainstem responses but robust cortical responses suggest abnormal subcortical encoding. With deaf users of CIs, a cortical measure indicating possible sites of dysfunction (e.g. primary auditory cortex or frontal cortex) could provide clinicians with optimal rehabilitation strategies (e.g. CI programming or cognitive training, respectively). These results may also provide a reference framework for a cortically driven CI to reduce listening effort, similar to the growing field of cognitively controlled hearing aids^68^.

## Methods

The current study follows a previous study^12^ that examined digits in noise speech perception in normal hearing adults. The stimuli and recordings were identical and described only briefly below.

### Participants

Ten adult cochlear implant participants (6 females; mean age: 49.5 years, range: 23–74 years) were recruited through Cincinnati Children’s Hospital Medical Center, according to an Institutional Review Board. All experimental protocols used in this study were approved by the Cincinnati Children’s Hospital Medical Center Institutional Review Board (Study number 2013-0105). All the methods used in this study were performed in accordance to the guidelines and regulations outlined in the Cincinnati Children’s Hospital Medical Center Institutional Review Board (Study number 2013-0105).

Participants had no clinically significant neurological or mental health issues. Participants received a monetary incentive and provided informed consent. All CI users had at least one-year CI experience prior to testing, a summary of the patient demographics is shown in Table 1.

**Table 1:** Clinical features of the CI users.

CI user	Age (years)	Gender	CI side	Stimulated ear	Duration of deafness (year)	CI use (year)	Device/Processor	Processing strategy	Etiology of hearing loss
1	30	M	Bilateral	Right	4	1	Nucleus/CI24RE	ACE	Unknown
2	32	F	Bilateral	Right	20	9	Nucleus/CI24RE	ACE	Congenital
3	37	F	Bilateral	Right	20	11	Nucleus/CI24RE	ACE	Unknown
4	45	F	Bilateral	Right	37	4	Nucleus/CI512	ACE	Unknown
5	45	F	Bilateral	Right	38	10	Nucleus/CI24RE	SPEAK	Unknown
6	56	F	Right	Right	15	2	Nucleus/CI24RE	ACE	Unknown
7	59	M	Bilateral	Right	11	1	Nucleus/CI24RE	ACE	Noise induced
8	60	M	Bilateral	Right	20	6	Nucleus/CI24RE	ACE	Genetic
9	63	F	Bilateral	Right	35	3	Nucleus/CI512	ACE	Genetic
10	68	M	Left	Left	22	2	Med EL/Opus 2	FSP	Genetic

### Stimuli and procedure

#### Digits

Procedures and rationale for recording, equalizing, and homogenizing the speech and noise stimuli have been presented in detail previously^12,31,69^. Briefly, all speech stimuli were recorded from a female talker of standard American English. The speech stimuli consisted of monosyllabic digits 0 to 9 (excluding the disyllabic 7), where the “0” was pronounced “Oh” (/oʊ/). Measured digit durations varied from 434–672 ms (SD 57 ms). The process created 27 unique digit files (9 digits, for each of the three digit positions). The long-term average speech spectrum for all 9 digits was mixed to create spectrally matched noise maskers.

A final step was a stimulus homogenization procedure^31,69^ that equated the audibility of the digits in noise for each digit and for each position. These steps were necessary because the speech reception threshold (SRT) performance measure of the DIN is based on equal audibility of all digits^12^.

#### Digits in Noise Test (DIN)

A customized Matlab program was designed to present the triplet digits in noise in successive trials, enabling the estimation of SRT, defined as the SNR yielding 50% correct identification for each set of three digits^69^ A graphical user interface (GUI) resembling a telephone touch key pad (i.e., 3 rows and 3 columns for digits 1 to 9 and bottom middle for the 0 digit; see Fig. 1a in Dimitrijevic and colleagues^12^) was incorporated for user response after stimulus presentation. The user initiated the beginning of the test and heard the noise masker, then the carrier phrase “The numbers”. The first digit occurred 1.5 seconds later, followed by the second and third digits (ISI = 1195 ms; onset to onset). The noise masker was turned off 1.5 seconds after the offset of the third digit. The entire stimulus (noise and masker) lasted 6 seconds. The GUI then allowed the participant to indicate which digits were heard. Signal to noise ratio (SNR) of successive triplets was varied adaptively from an initial level of +2 dB. Trials following a correct response (all three digits) had a reduced SNR by 2 dB (noise constant, digit amplitudes reduced). Incorrect responses were followed by an increased SNR, also by 2 dB. Twenty-five trials were presented and the average SNR over the last 11 trials was the SRT. All sounds were presented through a single loudspeaker at 0° azimuth 1.5 meters in front of the subject.

#### EEG

Electrophysiological recordings were performed after the behavioral SRT determination. EEG recordings used the same stimuli as behavioral testing, except that no introductory phrase was used and all trials had the same SNR. Two listening tasks were used; ‘passive’ listening, where the participants were instructed to ignore any sounds while they watched a closed caption and silent movie of their choice, and ‘attentive’ listening, where participants fixated a white cross on an otherwise blank computer screen. In the attentive listening task, rather than responding with the GUI, the participant verbally reported heard digits. The experimenter noted the perceived digits and then initiated the next trial. Signal SNR was initially set to the previously measured SRT (performance close to 50% for all three digits, see below). However, pilot behavioral data showed that participants perform better (lower SRTs) with repeated testing, as previously reported for the DIN, but only between the first and second blocks of trials^30^. Because we aimed to have roughly an equal number of correct and incorrectly identified trials (100 each) during EEG testing, we adopted an adaptive threshold approach, as above, using 2 blocks of 25 trials (50 trials), but with a starting SNR 2 dB below the previous behavioral SRT. Recordings were performed in 8 blocks (4 active and 4 passive) of 25 trials yielding 200 total trials. Participants took short breaks after each block. The attentive task always occurred first, followed by the passive listening task. The stimuli presented in the passive blocks were identical to that of the active blocks.

The electrophysiological data were collected using a 64-channel actiCHamp Brain Products recording system (Brain Products GmbH, Inc., Munich, Germany). An electrode cap was placed on the scalp with electrodes placed at equidistant locations^70^. The infracerebral cap used covers a larger area than is typical in a 10–20 system. The reference channel was located at vertex (Cz) while the ground electrode was located on the midline 50% of the distance to nasion. Continuous data were digitized at 1000 Hz and stored for offline analysis.

#### Self-report measures of listening effort

After each recording block CI users were asked to rate listening effort on a scale of 1–10 using the NASA Task Load Index^35^. Specifically, the participants indicated their effort level on a 10 point scale to the question: “How hard did you have to listen to accomplish your level of performance in that block?”, the same approach has been used in an earlier EEG study of listening effort^10^. Listening effort was not measured in the first two participants, leaving a sample size of 8.

### Data Processing

#### Preprocessing

The electrophysiological data were first processed using Brain Vision Analyzer ver. 2.0 (Brain Products GmbH, Inc., Munich, Germany). Data were high-pass filtered (0.1 Hz) to remove baseline drifts and down sampled to 250 Hz. Visual inspection and manual sorting of the data included removal of extreme stereotypical artifacts related to subject movement (exceeding 500 mV). Independent component analysis (ICA), as implemented in Brain Vision Analyzer (identical algorithm to EEGLAB^71^), was applied to reduce ocular and cardiac artifacts. Cochlear implant artifacts were identified by observing components with a centroid of activation on the CI side and the time course of ICA activity as we have described previously^72^.

Time-frequency analysis: Data were average referenced and segmented into epochs −1500 to 7000 ms relative to speech masker onset. All time-frequency analyses were performed in BESA 6.0 (Brain Electrical Source Analysis, GmbH, Germany) using 2 Hz frequency resolution across the epoch. BESA uses a two-step complex demodulation for time-frequency analysis described in detail in Hoechstetter and colleagues^37^. Changes in spectral power were quantified as a percent change from baseline (post-stimulus – pre-stimulus/pre-stimulus) x 100.

Brain source analysis: After the time-frequency analysis, a DICS beamformer^34^, as implemented in BESA was applied to a time-frequency region of interest. The choice of the time-frequency region of interest was based on the condition specific grand mean time-frequency analysis. The BESA DICS beamformer was implemented for the speech-brain coherence where phase coherence between a reference signal (low-pass filtered triple digit speech signal) and brain source were examined across trials in the 2–5 Hz range. A similar approach has been previously described in participants listening to vocoded speech^73^.

Statistical analysis: All statistical analyses were performed in BESA Statistics 2.0 in a similar manner to that previously described^70^. Differences between conditions were assessed by performing a paired t-test in source space and then corrected for multiple comparisons using Monte-Carlo resampling techniques^36^. Clusters of voxels with p-values of less than 0.05 were considered significant. Correlations between brain activity and measures of listening effort were implemented in the “Correlation” option in BESA Statistics 2.0. This procedure provides a correlational measure of brain activity associated with behavioral measure (e.g., listening effort). The source activity (e.g., alpha ERS/ERD over a particular time window) in each participant was performed. A single behavioral measure was then used to correlate brain activity in each voxel across all participants. This process yields a correlation value for each voxel. Corrections for multiple comparisons using Monte-Carlo resampling techniques^36^.

## Supplementary information

Supplementary data analysis
