# Supplementary material for: Neural indices of listening effort in noisy environments
Source: Sci Rep. 2019 Aug 2;9:11278. doi: 10.1038/s41598-019-47643-1 (PMC6677804; doi:10.1038/s41598-019-47643-1)
Supplement: Supplementary file 1 — Supplementary data analysis [file 41598_2019_47643_MOESM1_ESM.pdf]

Supplementary Material

Neural indices of listening effort in noisy environments.

Andrew Dimitrijevic[1,2,3*], Michael L Smith[1,9], Darren S Kadis[4,5,6], David R Moore[1,7,8]

1. Communication Sciences Research Center, Cincinnati Children's Hospital Medical Center
2. Department of Otolaryngology, Head and Neck Surgery, Sunnybrook Health Sciences Centre, Toronto, ON
3. Department of Otolaryngology, Head and Neck Surgery, University of Toronto, Toronto, ON
4. Division of Neurology, Cincinnati Children's Hospital Medical Center, Cincinnati OH
5. Pediatric Neuroimaging Research Consortium (PNRC), Cincinnati Children's Hospital Medical Center, Cincinnati OH
6. College of Medicine, Department of Pediatrics, University of Cincinnati, Cincinnati OH
7. Department of Otolaryngology, College of Medicine, University of Cincinnati, Cincinnati OH
8. Manchester Centre for Hearing and Deafness, University of Manchester, Manchester, UK
9. Department of Speech and Hearing Sciences, University of Washington, Seattle, WA.

* Correspondence to: andrew.dimitrijevic@sunnybrook.ca

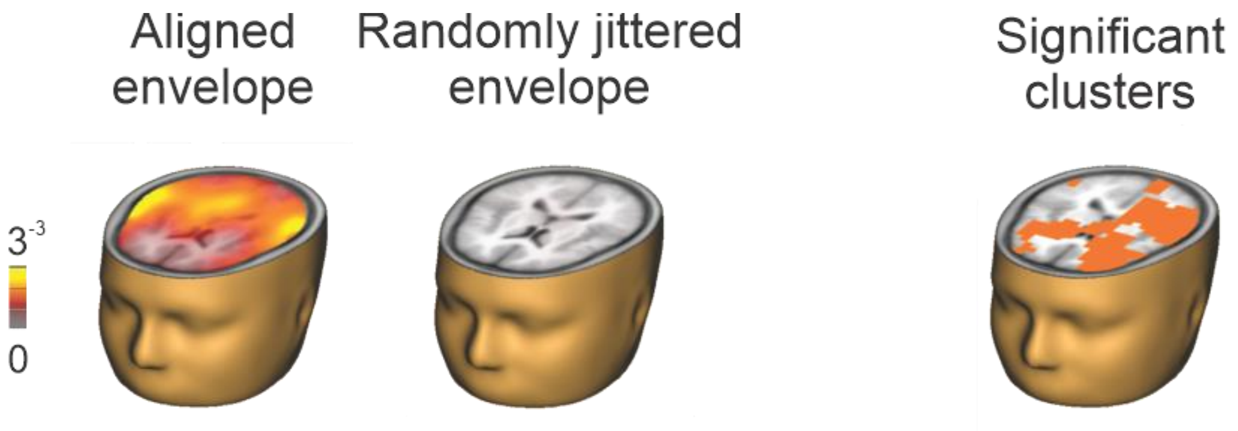

Figure S1. Significant coherence in the 2-5 Hz range to the speech envelope. In this analysis, the speech envelope was randomly jittered in the 0-6 sec range for each trial in each individual using a customized Matlab script. The DICS coherence was then again computed across all subjects on this randomly jittered condition and compared to the normal aligned envelope condition (as in Figures 3 and 4). Note that very low coherence is observed in the random jitter condition. A paired t-test comparing aligned versus random envelopes indicated that significantly greater coherence occurs in the left auditory temporal regions for the aligned envelopes.
